# Analysis of Local Recurrence After Robotic-Assisted Total Mesorectal Excision (ALRITE): An International, Multicentre, Retrospective Cohort

**DOI:** 10.3390/cancers17060992

**Published:** 2025-03-15

**Authors:** Ritch T. J. Geitenbeek, Rauand Duhoky, Thijs A. Burghgraef, Guglielmo Niccolò Piozzi, Shamsul Masum, Adrian A. Hopgood, Quentin Denost, Ellen van Eetvelde, Paolo Bianchi, Philippe Rouanet, Roel Hompes, Marcos Gómez Ruiz, Jim Briggs, Jim S. Khan, Esther C. J. Consten, on behalf of the ALRITE Study Group

**Affiliations:** 1 Department of Surgery, University Medical Center Groningen, University of Groningen, 9713 GZ Groningen, The Netherlands; r.t.j.geitenbeek@umcg.nl (R.T.J.G.);; 2 Department of Surgery, Meander Medical Center, 3813 TZ Amersfoort, The Netherlands; 3 Department of Colorectal Surgery, Portsmouth Hospitals University NHS Trust, Portsmouth PO6 3LY, UKguglielmo.piozzi@porthosp.nhs.uk (G.N.P.); 4 Faculty of Technology, University of Portsmouth, Portsmouth PO1 3HE, UK; adrian.hopgood@port.ac.uk; 5 School of Electrical and Mechanical Engineering, University of Portsmouth, Portsmouth PO1 3HE, UK; 6 Bordeaux Colorectal Institute, Clinique Tivoli, 33300 Bordeaux, France; 7 Department of Surgery, Universitair Ziekenhuis Brussel, 1090 Jette, Belgium; 8 General Surgery Unit, Department of Health Sciences (DISS), University of Milan San Paolo Hospital, 20142 Milan, Italy; 9 Surgery Department, Montpellier Cancer Institute (ICM), Université Montpellier, 34090 Montpellier, France; 10 Department of Surgery, University Medical Center Amsterdam, University of Amsterdam, 1105 AZ Amsterdam, The Netherlands; 11 Department of Surgery, Amsterdam Cancer Center, 1081 HV Amsterdam, The Netherlands; 12 Department of Surgery, Marqués de Valdecilla University Hospital, 39008 Santander, Spain; 13 Valdecilla Biomedical Research Institute (IDIVAL), 39011 Santander, Spain; 14 Centre for Healthcare Modelling and Informatics, University of Portsmouth, Portsmouth PO1 3HE, UK; 15 Faculty of Health Sciences, University of Portsmouth, Portsmouth PO1 3HE, UK

**Keywords:** total mesorectal excision, rectal cancer, artificial intelligence, prediction models, robot-assisted surgery

## Abstract

Background/Objectives: Rectal cancer is a major global health issue with high morbidity and mortality rates. Local recurrence (LR) significantly impacts patient outcomes, decreasing survival rates and often necessitating extensive secondary treatments. While robot-assisted total mesorectal excision (R-TME) is becoming a preferred method for rectal cancer surgery due to its improved precision and visualisation, long-term data on LR and predictors of recurrence remain limited. This study aims to determine the 3-year LR rate following R-TME and to identify predictors of recurrence to enhance patient selection and the personalisation of treatment. Methods: This retrospective international multicentre cohort study included 1039 consecutive rectal cancer patients who underwent R-TME between 2013 and 2020, with a minimum of 3 years of follow-up. Data from tertiary colorectal centres in the United Kingdom, the Netherlands, Spain, France, Italy, and Belgium were analysed. Potential predictors of LR were identified using backward elimination, and four machine learning models were evaluated for predicting LR. Results: The 3-year LR rate was 3.8%. Significant predictors of LR included advanced clinical M-staging, length of the hospital stay, postoperative ileus, postoperative complications, pathological N-staging, the completeness of resection, and the resection margin distance. The eXtreme Gradient Boosting model performed best for LR prediction, with a final accuracy of 77.1% and an AUC of 0.76. Conclusions: R-TME in high-volume centres achieves low 3-year LR rates, suggesting that robot-assisted surgery offers oncological safety and advantages in rectal cancer management. This study underscores the importance of surgical precision, patient selection, and standardised perioperative care, supporting further investment in robotic training to improve long-term patient outcomes.

## Simple Summary

Rectal cancer is a common and severe disease, and if the cancer recurs after surgery, it can significantly affect a patient’s health and chances of survival. Recent advances in robotic surgery have enabled more precise operations for rectal cancer, potentially lowering the risk of loco-regional recurrence. However, there is limited information on how often rectal cancer loco-regional recurrence occurs after robotic surgery and what factors might predict recurrence. This study examined the rate of loco-regional cancer recurrence within three years for patients who had undergone robot-assisted surgery and identified the key factors that may have increased the risk. By understanding these factors, we can better select patients who are most likely to benefit from robotic surgery and improve their follow-up care after surgery. These findings could help doctors and researchers to make more informed decisions to improve the long-term health outcomes for rectal cancer patients.

## 1. Introduction

Rectal cancer is one of the most prevalent malignancies worldwide, contributing significantly to cancer-related morbidity and mortality [1,2]. Local recurrence (LR) poses a considerable threat to patient outcomes due to its association with increased morbidity, decreased survival, and the need for aggressive secondary treatments [3].

Advances in surgical techniques, such as total mesorectal excision (TME) and minimally invasive approaches, have revolutionised the management of rectal cancer by improving local cancer control and reducing recurrence rates [3,4]. Among these innovations, robot-assisted surgery has gained traction due to its enhanced precision and superior visualisation. Recent meta-analyses suggest that robot-assisted surgery may reduce circumferential resection margin involvement and increase the likelihood of achieving complete oncological resection compared to conventional techniques [5,6,7]. However, much of the evidence is derived from small-scale randomised controlled trials (RCTs) and observational studies, which are often limited by small sample sizes, short follow-up periods, and variability in experience with surgical techniques, reducing the strength and generalizability of the evidence.

The recently published REAL RCT reported significantly higher rates of complete mesorectal excision with robot-assisted TME (R-TME) than with laparoscopic surgery [8]. These improved oncological resections may lead to better long-term oncological outcomes, but crucial long-term data on LR rates following robot-assisted surgery remain scarce. No RCTs, including the REAL trial [8], have yet reported on LR outcomes, leaving a significant gap in our understanding of the oncological efficacy of R-TME over time. Additionally, while several predictors of LR have been established in traditional surgical approaches, their applicability to robot-assisted procedures has not been thoroughly explored [9,10].

To address these gaps, this study aims to evaluate the LR rates in rectal cancer patients undergoing robot-assisted surgery. Additionally, we seek to identify potential predictors of LR in this population. By identifying the critical predictors of LR, this study could refine patient selection for robot-assisted surgery and optimise postoperative management strategies, ultimately contributing to personalised treatment approaches that improve the long-term outcomes and survival rates for rectal cancer patients.

To achieve these objectives, this retrospective international cohort study analysed LR rates and associated predictors in rectal cancer patients treated with robot-assisted surgery.

## 2. Methods

### 2.1. Study Design

This international, retrospective, multicentre cohort study analysed the three-year LR of rectal cancer patients treated with robot-assisted low anterior resections (LAR) from January 2013 to January 2020 with a minimum of three years of follow-up.

The data were sourced from tertiary referral centres specialised in colorectal cancer across the United Kingdom, the Netherlands, Spain, France, Italy, and Belgium. However, not all centres contributed data continuously throughout the entire study period. Instead, the data collection periods varied between centres, depending on when robotic surgery programmes were established and when each centre began contributing to this study. All centres maintained prospective research databases, with data collected from expert robot-assisted surgeons who had surpassed the learning curves for their respective techniques at the time of study enrolment, which was defined as having performed 35+ procedures [11]. All cases were reviewed in multidisciplinary team meetings and treated according to local and national guidelines.

A protocol was composed before the study’s initiation, and ethical approval was acquired (IRAS 310282). Ethical approval was also obtained at each participating centre according to their institutional and national guidelines, and this study was conducted following the “strengthening the reporting of observational studies in epidemiology guidelines for observational studies” (STROBE) guidelines (Supplementary S1) [12].

### 2.2. Patients

The inclusion criteria were as follows: (1) patients aged ≥ 18 years; (2) patients diagnosed with a rectal tumour located within 12 cm from the anorectal junction (ARJ) on magnetic resonance imaging (MRI); and (3) patients who underwent a curative robot-assisted proctectomy with partial or complete TME between 2013 and 2020 in an elective setting. The 12 cm distance from the ARJ on MRI was used, as over half of all tumours above this cut-off have been shown to consist of rectosigmoid cancers according to the anatomical landmark of the sigmoid take-off [13]. Unfortunately, data on the sigmoidal take-off was not consistently available for all participating centres, and thus we were unable to use this anatomical landmark to define the tumour location. The exclusion criteria were as follows: (1) patients undergoing abdominoperineal excision of the rectum (APR); (2) patients undergoing rectal cancer surgery as part of an extended procedure; and (3) patients who had palliative or emergency resections.

The preoperative diagnostics comprised patient demographics, tumour histology, and cancer staging, including chest, abdomen, and pelvis computed tomography (CT) scanning and pelvic MRI. Depending on patient and tumour characteristics, various neoadjuvant and adjuvant therapies were utilised. Commonly used neoadjuvant therapies included neoadjuvant chemoradiation therapy (NACRT), only short-course radiotherapy (SCRT), or total neoadjuvant therapy (TNT) (with the addition of induction or consolidation chemotherapy with NACRT or SCRT), generally using FOLFOX or FOLFIRINOX. Patients underwent SCRT or NACRT after multidisciplinary team discussion, with clinical restaging and surgery performed afterward. NACRT regimens included either short-course radiotherapy (25 Gy in 5 fractions) or long-course chemoradiotherapy (45–50 Gy in 25 fractions) with concurrent chemotherapy (CAPOX or FOLFOX). Clinical and oncological outcomes were recorded in the patient notes.

### 2.3. Surgical Procedure

The surgical approach was determined by surgeon preference, patient input, and platform availability. All robot-assisted procedures were performed using the da Vinci Si/X/Xi systems (Intuitive Surgical, Sunnyvale, CA, USA). The patients underwent mechanical bowel preparation before surgery, according to protocols [14]. All surgical procedures adhered to the standardised protocols for TME or partial mesorectal excision (PME, also defined as tumour-specific mesorectal excision). TME requires the removal of the entire mesorectum down to the pelvic floor. PME was performed for the resection of rectal tumours located more than 5 cm proximal to the pelvic floor. This technique obviates the need for complete distal mesorectal excision to achieve oncological adequacy and is associated with a reduced risk of anastomotic leakage, attributable to the improved vascularisation of the rectal stump. Lymph node dissection at the origin of the inferior mesenteric artery was standard, and hypogastric nerve preservation was attempted where feasible. Decisions regarding open conversion and the formation of diverting stomas were left to the surgeon’s discretion. Postoperatively, patients followed standardised enhanced recovery protocols, with clinical and oncological outcomes documented meticulously. Postoperative management followed standardised enhanced recovery protocols and national follow-up guidelines.

### 2.4. Outcomes and Definitions

This study’s primary focus was to determine the incidence of LR after R-TME and to identify possible predictive factors. Secondly, this study aimed to develop a machine-learning-based preoperative predictive model for LR. LR was defined as any tumour mass in the pelvis that was either pathologically proven or suspected of recurrence on radiological imaging. The included variables are described in-depth in Supplementary S2.

### 2.5. Statistical Analysis

All statistical analyses were conducted using the Python (version 3.11.11) language in a Google Colaboratory environment (“sklearn”, “pandas”, “numpy”, “imblearn”, “shap”, “lime”, and “pickle”) or using R version 4.3.0 (R Core Team, 2023, Vienna, Austria). The percentages shown in the results pertain to the data available after missing values were removed. Categorical variables were presented as absolute case counts and percentages, and continuous variables were summarised by their means and standard deviations (SDs) for normally distributed data, or medians and interquartile ranges for non-normally distributed data.

Baseline characteristics were summarised for each group using appropriate descriptive statistics. Missing data were handled using a K-Nearest Neighbour (KNN) imputer from the sklearn “impute” package. Data were first analysed with a backwards elimination model using an ExtraTreesClassifier to identify the factors that significantly correlated with LR. After that, four types of prediction models (Random Forest Classifier (RFC), Long Short-Term Memory (LSTM), Decision Tree Classifier (DTC), and eXtreme Gradient Boosting (XGB)) were explored for building two prediction models with LR as a binary outcome (one with all variables and one with only the factors identified during the backwards elimination step).

Approximately 10% of the dataset was excluded from any of the training or testing phases to serve as an external validation. The remaining data were split into training (70%) and testing (30%) and assessed through performance metrics on the external validation dataset using Receiver Operator Characteristic (ROC) Area Under the Curve (AUC) scores, accuracy, sensitivity, and specificity. Bootstrapping was performed to reinforce the model in the random train–test split, and only performance metrics for the external/final validation stage were considered to tackle overfitting. Leave-One-Out cross-validation was also tested for the model.

All models underwent undersampling to tackle the innate class imbalances. Modelling was subsequently performed in three stages: one with only random undersampling, one with random undersampling and oversampling using the “Synthetic Minority Oversampling Technique” (SMOTE) for the training-and-testing phase, and one with random over- and undersampling using SMOTE for both the training-and-testing and validation phase.

SHapley Additive exPlanations (SHAP) and Local Interpretable Model-Agnostic Explanations (LIME) were used for explainable AI through both general model explanations and the explaining of individual examples. A binary “imputation check” variable was added before any training/testing to ensure that imputation did not affect the model. Where both the imputation check variable and the original variable were considered relevant to the prediction model in the explainable AI assessment, this variable was dropped from the prediction model and the model was retrained.

## 3. Results

### 3.1. Overall Results

A total of 1039 patients were included in this study. The median age of the cohort was 68 years, with a median BMI of 25.92 kg/m². The majority of patients were male (64.4%). On preoperative staging, the majority of patients had cT3 (62.0%) and cN+ (53.4%) tumours. Furthermore, the median tumour distance from the ARJ was 7 cm, and 41.8% of patients had mesorectal fascia (MRF) involvement.

Neoadjuvant therapy was administered to 59.1% of patients, with chemoradiation (58.3%) being the most commonly used regimen. The pathological assessment of the resected specimens revealed that 87.7% of patients had a complete mesorectal excision, while 4.0% had a positive circumferential resection margin (CRM). Postoperatively, 46.8% of patients developed complications within 30 days, and 29.5% of the cohort received adjuvant chemotherapy. The overall 3-year LR rate was 3.8% (*n* = 41), and the descriptive variables are displayed in Table 1.

Backward elimination (BE) using an ExtraTreesClassifier identified seven out of fifty tested variables to be statistically significant risk factors for LR: clinical M-staging, length of hospital stay, postoperative ileus, Clavien–Dindo classification of any postoperative complication, pathological N-staging, pathological resection staging, and pathological distance to the resection margin (all *p* < 0.05). Model testing was performed on both the full dataset and a “subset” containing only LR and the seven statistically significant risk factors.

### 3.2. Prediction Modelling Without over- and Undersampling

For both the complete dataset and the subset, XGB proved the best at predicting, with 500 iterations of bootstrapping for the complete dataset and 1100 iterations for the subset. Without oversampling, the complete dataset had a validation accuracy of 55.4%, a sensitivity of 75.0%, a specificity of 52.1%, and an AUC of 0.61. The subset performance metrics were a 53.6% accuracy, a 50.0% sensitivity, a 54.2% specificity, and an AUC of 0.64.

### 3.3. Prediction Modelling with Oversampling Only in Training-And-Testing

With oversampling in only the training-and-testing phase, the complete dataset performed with 87.5% accuracy, 50.0% sensitivity, 93.8% specificity, and an AUC of 0.68. The subset performance metrics were 71.4% accuracy, 25.0% sensitivity, 79.2% specificity, and an AUC of 0.73.

### 3.4. Prediction Modelling with Oversampling in Training-And-Testing and Validation

**Figure 1:**
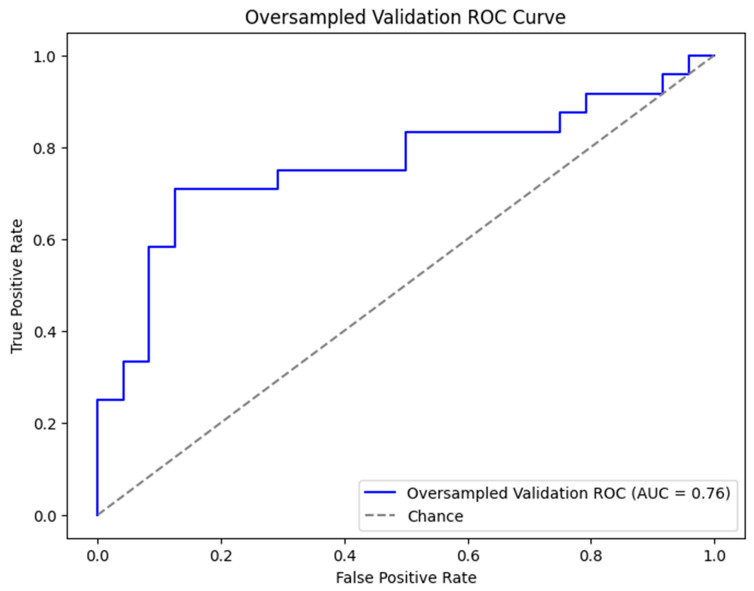
ROC curve for XGB (eXtreme Gradient Boosting) prediction model with all variables included and oversampling in both training-and-testing (500 iterations of bootstrapping) and validation phases. ROC curve and AUC are metrics for demonstrating performance of model. Abbreviations: XGB, eXtreme Gradient Boosting; ROC, Receiver Operator Characteristic; and AUC, Area Under the Curve.

**Figure 2:**
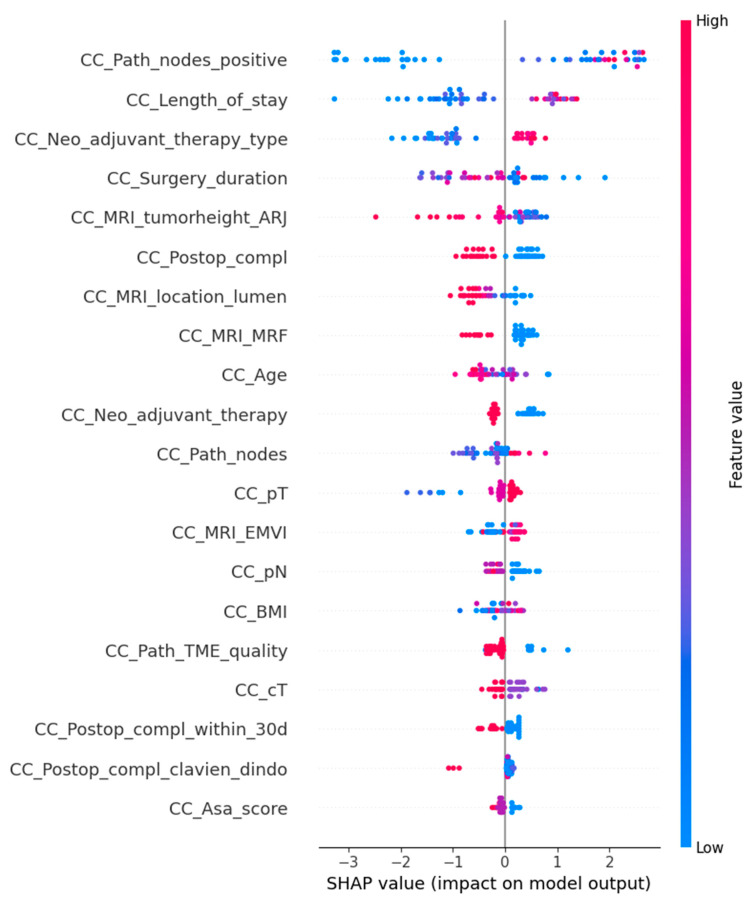
SHapley Additive exPlanations (SHAP) is an explainable AI model for the general model explanation of the XGB (eXtreme Gradient Boosting) prediction model. The feature value demonstrates the value of that variable, with blue highlighting the lower end of the scale and red highlighting the higher end of the scale. The variables are named on the left, and the dots represent the SHAP value that each variable has on the total model output, with pooled dots showing a more uniform and significant contribution to the model.

**Figure 3:**
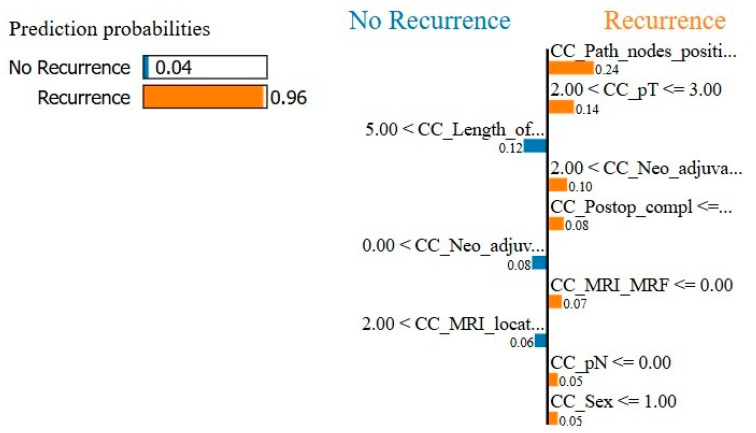
Local Interpretable Model-Agnostic Explanations (LIME) is an explainable AI model for the local explanation of the XGB (eXtreme Gradient Boosting) prediction model. The left is the total prediction probability of the model, which should approximately correspond with the incidence of the output variable. The right shows an example of a random prediction and which variables contributed in which way to the model decision.

For the final model with oversampling in both the training-and-testing and validation phases, the complete dataset performed with 77.1% accuracy, 66.7% sensitivity, 87.5% specificity, and an AUC of 0.76 (Figure 1). The subset performance metrics were 64.6% accuracy, 58.3% sensitivity, 70.8% specificity, and an AUC of 0.75. The explainable AI for the final model is shown in Figure 2 and Figure 3.

## 4. Discussion

The recurrence of rectal cancer remains a significant factor in patient prognosis, with direct implications for survival and quality of life [1,2]. This multicentre, retrospective study demonstrated a 3-year LR rate of 3.8% following R-TME, identifying key predictive factors: advanced clinical M-staging, a prolonged hospital stay, postoperative ileus, the Clavien–Dindo classification of any postoperative complication, pathological N-staging, the completeness of resection, positive circumferential resection margins, and the distance of distal resection margin. These predictors underscore the complexity of managing rectal cancer in patients and highlight the importance of precision in surgical resection for optimal outcomes.

The 3.8% 3-year LR rate observed in this study represents a high-quality surgical performance. Notably, this rate compares favourably with historical data: the ROLARR trial [10], for instance, reported higher 3-year LR rates for laparoscopic (6.0%) and robot-assisted TME (6.8%) approaches, likely influenced by early-stage surgeon learning curves. The lower LR rate in our study may reflect advancements in robotic technology, which enhances visualisation, dexterity, and precision—particularly beneficial in the anatomically challenging pelvis. Robotic platforms, as demonstrated in the REAL trial [8] and a previous meta-analysis [5], facilitate a higher rate of complete mesorectal excisions and reduce positive circumferential resection margins, which are crucial for minimising LR risk. However, it is important to acknowledge that the low LR rates observed in our study may also reflect the inclusion of data from highly experienced centres, which could introduce a selection bias and limit the generalizability to broader surgical settings. Nevertheless, this study’s lack of a direct comparison group precludes definitive conclusions on the potential superiority of R-TME over laparoscopic TME.

When comparing our findings with non-randomised studies on R-TME, variations in follow-up duration, patient selection, and surgeon experience must be considered. For instance, the studies by Patriti et al. [15] and Kwak et al. [16] observed a 0% and 1.8% recurrence rate following R-TME. However, these studies only had a 12-month follow-up, limiting the assessment of long-term recurrence. Popescu et al. [17] reported LR rates of 5.2% for robot-assisted surgery. These rates are higher than the current study, despite their research excluding patients who underwent Hartmann’s procedure, which has been shown to have worse long-term oncological outcomes [18]. Notably, these earlier studies occurred during the initial adoption of robotic surgery, suggesting that the low LR rate in the present study may reflect both improvements in surgical technique as well as advancements in preoperative staging, neoadjuvant therapy, and perioperative care.

While one might argue that limiting the analysis to preoperative variables could be more applicable to clinical practice, this study attempted such a sub-analysis but failed to yield any predictive models of utility. The likely explanation is that the relationship between preoperative variables and the SR outcome was too weak for the ML model to reliably train within this cohort. However, the inclusion of intraoperative, perioperative, and short-term postoperative data augmented the dataset, enabling the ML model to leverage its predictive capabilities. Future research focusing solely on preoperative variables may be valuable but will need to overcome substantial challenges in acquiring large-scale, high-quality data with adequate follow-up.

This study’s identification of factors such as advanced pN-staging and resection margin distance as predictors of LR aligns with the established literature linking these pathological features with poor prognosis. For example, Kozu et al. [9] noted the association of pN+ status with aggressive tumour biology, supporting the hypothesis that precise surgical techniques can mitigate recurrence in high-risk cases. Although the prognostic significance of resection margin distance has been debated recently, our findings suggest that it may be associated with LR risk. One possible explanation is that a narrower margin may indicate microscopic residual disease or an increased likelihood of tumour cell dissemination in the surgical field, contributing to local recurrence. Additionally, findings indicating that postoperative ileus and extended hospital stays correlate with increased LR risk highlight the role of surgical quality and patient recovery in achieving optimal oncologic outcomes. One plausible explanation for the observed association between extended hospital stays and increased LR risk is the potential occurrence of (early) anastomotic leakage, which may have prolonged recovery times. Previous studies have hypothesised that anastomotic leakage could contribute to higher recurrence rates through several mechanisms. For instance, research on peritoneal recurrence after colorectal cancer surgery suggests that viable cancer cells may spill into the peritoneal cavity during surgery, with the inflammatory response to surgical trauma enhancing tumour cell adherence and subsequent growth [19,20]. Another hypothesis is that the localised inflammatory reaction due to leakage upregulates the expression of the receptors associated with tumour cell adhesion, as demonstrated in rat models of surgical injury [21]. This reaction may not be confined to the localised tissue but could extend to the entire intra-abdominal cavity due to systemic inflammatory responses [22,23]. This might explain the association between anastomotic leakage and increased (intra-abdominal) recurrence rates. However, further research is needed to validate these hypotheses, particularly as they rely on the assumption that spilled tumour cells remain viable up to the time of leakage, which can occur anywhere from a few days to months after surgery. These findings emphasise the importance of both preoperative risk stratification and perioperative optimisation, not only to mitigate early postoperative complications but also to reduce the risk of local recurrence and improve long-term cancer control.

Interestingly, body mass index (BMI) was not significantly associated with LR in the present study, consistent with results from the ROLARR trial [10] and several non-randomised studies [24,25,26,27]. In contrast, evidence from other cohorts [9,28,29], including the Japanese multicentre study by Kozu et al. [9], associated higher BMI with worse LR rates in stage II/III rectal cancer. Notably, a previous study suggested a significant association between BMI and LR solely in the lower rectum [30], suggesting that having a higher BMI may have less influence on the surgeon’s ability to perform a clear resection in middle- and upper-rectal cancer. The current study also included middle- and high-rectal tumours, which may have contributed to finding no significant predictive value of BMI. In particular, laparoscopic surgery’s challenges with obesity may not be as pronounced in robotic platforms, which offer superior visualisation and mobility in confined pelvic spaces.

Preoperative MRI markers, including cT category, mrCRM, and mrEMVI, did not predict LR in the present cohort. A potential explanation for this finding is that postoperative pathological outcomes, such as pathological staging and resection margin status, were included as predictors in the multivariate analyses. As pathological staging is generally more accurate and reliable than preoperative MRI staging, it is likely that the inclusion of these postoperative variables diminished the significance of preoperative MRI markers in predicting LR. Sub-analyses with only the preoperative findings were attempted, but did not produce models of any significance, most likely due to a lack of training data for these ML models. Future analyses in larger cohorts with only preoperative variables may be beneficial for the development of further refined prediction models, but face severe logistical challenges in producing even larger international multicentre datasets with reliable data entry points.

The identified predictors align with the study by Kozu et al. [9], which also reported no association between these factors and LR. In contrast, prior studies have linked these factors with oncological outcomes [31,32,33,34]. This discrepancy could also be attributed to non-standardised MRI protocols and variations in image quality across participating centres. Moreover, the lack of association between MRI predictors and LR may reflect the inclusion of data from over a decade ago, when MRI availability and quality were more limited. These observations underscore the importance of standardised imaging protocols and advancements in imaging technology to enhance predictive accuracy and ensure consistency across centres.

The use of ML models in this study represents a novel approach to identifying patients at risk of LR after rectal cancer surgery. Although the predictive performances of the models were modest, with AUC values ranging from 0.61 to 0.76, these results highlight the potential of ML to complement traditional clinical assessments. Predictive models could, with further validation and refinement, be used to enhance preoperative risk stratification, allowing for tailored surgical planning and postoperative care. For instance, patients identified as high-risk could benefit from more intensive follow-up protocols or adjunctive therapies to mitigate recurrence risks. Additionally, the explainability features of the ML models provide actionable insights into key predictive factors, supporting a data-driven approach to decision-making.

This study’s strengths include its large-scale, multicentre design involving high-volume centres, enhancing its generalizability to tertiary care environments where surgeons have surpassed the robotic learning curve. However, this study’s retrospective design introduces inherent biases, such as variations in perioperative care and a lack of standardised preoperative imaging. The use of ML to build an LR prediction model in rectal cancer is another strength, as this has not yet been performed in the existing literature for surgical data, with previous studies being limited to regressive statistics [35,36,37]. The absence of a direct laparoscopic TME comparison and the limited five-year follow-up data constrain the interpretability of long-term outcomes. Future research should prioritise prospective, randomised trials directly comparing R-TME and laparoscopic TME, with standardised perioperative and imaging protocols to validate these findings and assess the true impact of robotic platforms on oncological precision.

## 5. Conclusions

This international, multicentre study demonstrates that R-TME performed in high-volume centres by experienced surgeons is associated with a low 3-year LR rate of 3.8%, supporting its oncological safety in rectal cancer surgery. Key predictors of LR include preoperative clinical staging, postoperative complications, and pathological staging, underscoring the importance of surgical quality and perioperative optimisation. However, the absence of a direct laparoscopic TME comparison limits definitive conclusions on the effectiveness of R-TME.

This study also highlights the potential role of ML models in LR prediction, though their clinical utility remains limited. Future prospective trials comparing robotic and laparoscopic TME with standardised protocols are needed to better define the long-term oncologic impact of robotic platforms.

## Figures and Tables

**Table 1:** Descriptive variables used for LR prediction model.

Descriptive Variables Used for LR Prediction Model				
	**Level**	**Full Dataset**	**No LR**	**LR**
*n*		1039	984	41
Site (%)	The Netherlands	363 (34.9)	346 (35.2)	16 (39.0)
	France	190 (18.3)	179 (18.2)	9 (22.0)
	UK	148 (14.2)	143 (14.5)	3 (7.3)
	Spain	233 (22.4)	221 (22.5)	11 (26.8)
	Italy	74 (7.1)	66 (6.7)	1 (2.4)
	Belgium	31 (3.0)	29 (2.9)	1 (2.4)
Sex (%)	Male	669 (64.4)	633 (64.3)	27 (65.9)
	Female	370 (35.6)	351 (35.7)	14 (34.1)
Age at time of surgery in years (median [IQR])		68.00 [61.00, 75.00]	67.00 [60.00, 74.00]	67.00 [61.00, 75.00]
ASA score (%)	I	173 (16.7)	166 (16.9)	7 (17.1)
	II	675 (65.1)	638 (64.8)	27 (65.9)
	III	184 (17.7)	175 (17.8)	7 (17.1)
	IV	5 (0.5)	5 (0.5)	0 (0.0)
BMI (median [IQR])		25.92 [23.49, 29.06]	26.00 [23.50, 28.93]	25.41 [23.71, 27.93]
Diabetic comorbidity (%)	No	648 (89.3)	620 (89.6)	23 (79.3)
	Yes	78 (10.7)	72 (10.4)	6 (20.7)
Cardiovascular comorbidity (%)	No	461 (68.4)	440 (68.3)	18 (69.2)
	Yes	213 (31.6)	204 (31.7)	8 (30.8)
History of abdominal surgery (%)	No	519 (74.5)	493 (74.1)	23 (82.1)
	Yes	178 (25.5)	172 (25.9)	5 (17.9)
cT (%)	cT1	27 (3.6)	27 (3.8)	0 (0.0)
	cT2	223 (29.4)	213 (29.7)	8 (27.6)
	cT3	469 (62.0)	440 (61.3)	19 (65.5)
	cT4	38 (5.0)	36 (5.0)	2 (6.9)
cN (%)	cN0	300 (46.6)	291 (47.8)	7 (30.4)
	cN1	245 (38.0)	226 (37.1)	9 (39.1)
	cN2	99 (15.4)	92 (15.1)	7 (30.4)
cM (%)	cM0	704 (92.1)	671 (92.9)	22 (75.9)
	cM1	60 (7.9)	51 (7.1)	7 (24.1)
Distance from the ARJ on MRI in cm (median [IQR])		7.00 [5.00, 9.00]	7.00 [4.30, 9.00]	5.00 [2.88, 9.00]
Tumour location on MRI (%)	Anterior	107 (16.1)	106 (16.7)	1 (3.7)
	Circumferential	270 (40.5)	255 (40.2)	14 (51.9)
	Lateral	59 (8.9)	57 (9.0)	2 (7.4)
	Posterior	95 (14.3)	88 (13.9)	3 (11.1)
	Semi-circumferential	15 (2.3)	15 (2.4)	0 (0.0)
	Other	120 (18.0)	113 (17.8)	7 (25.9)
MRF on MRI (%)	MRF−	386 (58.2)	370 (58.7)	15 (53.6)
	MRF+	277 (41.8)	260 (41.3)	13 (46.4)
EMVI on MRI (%)	EMVI−	252 (50.8)	244 (51.2)	8 (44.4)
	EMVI+	244 (49.2)	234 (48.8)	10 (55.6)
Neoadjuvant therapy (%)	No	425 (40.9)	410 (41.7)	12 (29.3)
	Yes	614 (59.1)	574 (58.3)	29 (70.7)
Type of neoadjuvant therapy (%)	None	425 (41.0)	410 (41.8)	12 (29.3)
	Only chemo	10 (1.0)	9 (0.9)	1 (2.4)
	Short-course radiotherapy	90 (8.7)	86 (8.8)	3 (7.3)
	Long-course radiotherapy	2 (0.2)	2 (0.2)	0 (0.0)
	Chemoradiation	358 (34.6)	338 (34.4)	14 (34.1)
	Chemoradiation + chemo (TNT)	109 (10.5)	99 (10.1)	9 (22.0)
	Other	42 (4.1)	37 (3.8)	2 (4.8)
Conversion (%)	No	1002 (96.4)	950 (96.5)	38 (92.7)
	Yes	37 (3.6)	34 (3.5)	3 (7.3)
Anastomosis performed (%)	No	128 (12.4)	123 (12.5)	3 (7.3)
	Yes	908 (87.6)	858 (87.5)	38 (92.7)
Stoma placed (%)	No	310 (29.8)	301 (30.6)	8 (19.5)
	Yes	729 (70.2)	683 (69.4)	33 (80.5)
Drain placed (%)	No	197 (32.7)	190 (32.9)	7 (28.0)
	Yes	406 (67.3)	388 (67.1)	18 (72.0)
Intraoperative complication (%)	No	633 (96.3)	596 (96.3)	26 (96.3)
	Yes	24 (3.7)	23 (3.7)	1 (3.7)
Intraoperative perforation (%)	No	615 (98.7)	581 (98.6)	25 (100.0)
	Yes	8 (1.3)	8 (1.4)	0 (0.0)
Other intraoperative complication (%)	No	612 (99.2)	577 (99.1)	25 (100.0)
	Pancreas, liver, choledo, gallbladder	2 (0.3)	1 (0.2)	0 (0.0)
	Bladder requiring sutures	2 (0.3)	2 (0.3)	0 (0.0)
	Other	1 (0.2)	2 (0.3)	0 (0.0)
Operative time (median [IQR])		245.00 [195.00, 300.00]	243.00 [195.00, 300.00]	260.00 [182.00, 338.00]
pT (%)	pT0	79 (7.8)	78 (8.0)	0 (0.0)
	pT1	102 (10.0)	101 (10.3)	1 (2.4)
	pT2	312 (30.7)	301 (30.7)	8 (19.5)
	pT3	499 (49.1)	463 (47.2)	27 (65.9)
	pT4	25 (2.5)	21 (2.1)	4 (9.8)
pN (%)	pN0	667 (64.8)	645 (66.1)	14 (35.0)
	pN1	285 (27.7)	265 (27.2)	15 (37.5)
	pN2	78 (7.6)	66 (6.8)	11 (27.5)
pM (%)	pM0	732 (92.7)	695 (93.0)	28 (84.8)
	pM1	58 (7.3)	52 (7.0)	5 (15.2)
Lymph nodes harvested (median [IQR])		15.00 [11.00, 21.00]	15.00 [11.00, 21.00]	15.00 [9.00, 22.00]
Number of positive lymph nodes harvested (median [IQR])		0.00 [0.00, 1.00]	0.00 [0.00, 1.00]	1.00 [0.00, 3.75]
CRM (%)	CRM−	935 (96.0)	891 (96.5)	32 (84.2)
	CRM+	39 (4.0)	32 (3.5)	6 (15.8)
DRM (median [IQR])		10.00 [10.00, 30.00]	10.00 [10.00, 30.00]	10.00 [5.00, 20.00]
Completeness of resection (%)	Incomplete	24 (3.2)	23 (3.3)	0 (0.0)
	Nearly complete	67 (9.1)	61 (8.7)	6 (21.4)
	Complete	649 (87.7)	617 (88.0)	22 (78.6)
Pathological resection radicality (%)	R0	946 (95.6)	904 (96.3)	31 (79.5)
	R1	37 (3.7)	30 (3.2)	6 (15.4)
	R2	7 (0.7)	5 (0.5)	2 (5.1)
Pathological histology (%)	Adenocarcinoma	789 (92.3)	749 (92.8)	32 (86.5)
	Mucinous	54 (6.3)	48 (5.9)	4 (10.8)
	Signet cell	1 (0.1)	1 (0.1)	0 (0.0)
	Other	11 (1.3)	9 (1.1)	1 (2.7)
Pathological perforation (%)	No	489 (98.0)	466 (97.9)	22 (100.0)
	Yes	10 (2.0)	10 (2.1)	0 (0.0)
Postoperative complications (%)	No	544 (53.2)	514 (53.1)	21 (51.2)
	Yes	478 (46.8)	454 (46.9)	20 (48.8)
Postoperative complications within 30 days (%)	No	366 (58.5)	341 (57.8)	17 (65.4)
	Yes	260 (41.5)	249 (42.2)	9 (34.6)
Ileus (%)	No	525 (86.5)	498 (86.8)	19 (76.0)
	Yes	82 (13.5)	76 (13.2)	6 (24.0)
Wound infection (%)	No	566 (93.1)	534 (93.2)	23 (92.0)
	Yes	42 (6.9)	39 (6.8)	2 (8.0)
Bleeding (%)	No	804 (95.3)	757 (95.1)	36 (97.3)
	Yes	40 (4.7)	39 (4.9)	1 (2.7)
Other postoperative complications (%)	No	414 (68.7)	386 (68.2)	19 (73.1)
	Yes	54 (9.0)	50 (8.8)	3 (11.5)
	Infectious	8 (1.3)	7 (1.2)	1 (3.8)
	Neurologic	4 (0.7)	4 (0.7)	0 (0.0)
	Urological	21 (3.5)	21 (3.7)	0 (0.0)
	Other non-surgical	18 (3.0)	18 (3.2)	0 (0.0)
	Abscess	7 (1.2)	7 (1.2)	0 (0.0)
	Fascia dehiscence	1 (0.2)	1 (0.2)	0 (0.0)
	Bowel perforation	5 (0.8)	4 (0.7)	0 (0.0)
	Leakage of bladder/ureter	1 (0.2)	1 (0.2)	0 (0.0)
	Other surgical complication	45 (7.5)	45 (8.0)	0 (0.0)
	Pulmonary	13 (2.2)	11 (1.9)	2 (7.7)
	Cardiac	11 (1.8)	11 (1.9)	0 (0.0)
	Thromboembolic	1 (0.2)	0 (0.0)	1 (3.8)
Clavien–Dindo (%)	None	544 (53.2)	514 (53.1)	21 (51.2)
	I	117 (11.4)	111 (11.5)	6 (14.6)
	II	190 (18.6)	184 (19.0)	5 (12.2)
	IIIa	37 (3.6)	32 (3.3)	5 (12.2)
	IIIb	103 (10.1)	96 (9.9)	4 (9.8)
	IV	21 (2.1)	21 (2.1)	0 (0)
	V	10 (1.0)	10 (1.0)	0 (0)
Anastomotic leakage (%)	No	731 (85.0)	695 (85.0)	27 (87.1)
	Yes	129 (15.0)	123 (15.0)	4 (12.9)
Reintervention within 31 days (%)	No	919 (88.6)	870 (88.5)	38 (92.7)
	Yes	118 (11.4)	113 (11.5)	3 (7.3)
Readmission within 31 days (%)	No	924 (89.1)	876 (89.1)	36 (87.8)
	Yes	113 (10.9)	107 (10.9)	5 (12.2)
Length of stay (postoperative) (median (IQR))		7.00 [5.00, 11.00]	7.00 [5.00, 11.00]	11.00 [6.00, 17.00]
Adjuvant chemotherapy (%)	No	694 (70.5)	664 (70.8)	24 (61.5)
	Yes	291 (29.5)	274 (29.2)	15 (38.5)
Systemic recurrence (%)	No	852 (83.0)	825 (83.8)	26 (63.4)
	Yes	175 (17.0)	159 (16.2)	15 (36.6)
Local recurrence (%)	No	984 (96.0)	984 (100)	0 (0)
	Yes	41 (4.0)	0 (0)	41 (100)

Abbreviations: LR, local recurrence; IQR, interquartile range; ARJ, anorectal junction; ASA, American Society of Anesthesiologists; BMI, body mass index; cT, clinical tumour staging; cN, clinical nodal staging; cM, clinical metastatic staging; MRI, magnetic resonance imaging; MRF, mesorectal fascia; EMVI, extramural venous invasion; TNT, total neoadjuvant therapy; pT, pathological tumour staging; pN, pathological nodal staging; pM, pathological metastatic staging; CRM, circumferential resection margin; DRM, distal resection margin; EMVI, extramural venous invasion; IQR, interquartile range; MRF, mesorectal fascia; MRI, magnetic resonance imaging; *n*, total number of patients; UK, United Kingdom.

## Data Availability

Anonymised data can be made available from the corresponding author following reasonable request. In compliance with what has been agreed to in the consortium agreement and informed consent, anonymised data can be made accessible to other researchers if they comply with United Kingdom legislation and comply with any restrictions that the ethics committee might impose on the reuse. To do so, researchers would have to contact the ALRITE Steering Committee.

## Supplementary Materials

The following supporting information can be downloaded at: https://www.mdpi.com/article/10.3390/cancers17060992/s1, Supplementary S1: STROBE checklist; Supplementary S2: Variable descriptions. References [38,39] are cited in the Supplementary Materials.
