# Supplementary material for: Analysis of Local Recurrence After Robotic-Assisted Total Mesorectal Excision (ALRITE): An International, Multicentre, Retrospective Cohort
Source: Cancers (Basel). 2025 Mar 15;17(6):992. doi: 10.3390/cancers17060992 (PMC11940555; doi:10.3390/cancers17060992)
Supplement: Supplementary file 1 [file cancers-17-00992-s001.zip › cancers-3485566-supplementary.pdf]

Supplementary S1: STROBE checklist

STROBE Statement—checklist of items that should be included in reports of observational studies

	Item No.	Recommendation	Page No.	Relevant text from manuscript
**Title and abstract**	1	(*a*) Indicate the study's design with a commonly used term in the title or the abstract	1	
		(*b*) Provide in the abstract an informative and balanced summary of what was done and what was found	3	
**Introduction**				
Background/rationale	2	Explain the scientific background and rationale for the investigation being reported	4	
Objectives	3	State specific objectives, including any prespecified hypotheses	4	
**Methods**				
Study design	4	Present key elements of study design early in the paper	5	
Setting	5	Describe the setting, locations, and relevant dates, including periods of recruitment, exposure, follow-up, and data collection	5-7	
Participants	6	(*a*) *Cohort study*—Give the eligibility criteria, and the sources and methods of selection of participants. Describe methods of follow-up	5-7	
		(*b*) *Cohort study*—For matched studies, give matching criteria and number of exposed and unexposed	NA	
Variables	7	Clearly define all outcomes, exposures, predictors, potential confounders, and effect modifiers. Give diagnostic criteria, if applicable	5-7	

Data sources/ measurement	8*	For each variable of interest, give sources of data and details of methods of assessment (measurement). Describe comparability of assessment methods if there is more than one group	5-7
Bias	9	Describe any efforts to address potential sources of bias	5-7
Study size	10	Explain how the study size was arrived at	5-7

Continued on next page

Quantitative variables	11	Explain how quantitative variables were handled in the analyses. If applicable, describe which groupings were chosen and why	6-7
Statistical methods	12	(*a*) Describe all statistical methods, including those used to control for confounding	6-7
		(*b*) Describe any methods used to examine subgroups and interactions	6-7
		(*c*) Explain how missing data were addressed	6-7
		(*d*) *Cohort study*—If applicable, explain how loss to follow-up was addressed	6-7
		(*e*) Describe any sensitivity analyses	6-7
**Results**			
Participants	13*	(a) Report numbers of individuals at each stage of study—eg numbers potentially eligible, examined for eligibility, confirmed eligible, included in the study, completing follow-up, and analysed	Table 1
		(b) Give reasons for non-participation at each stage	Table 1
		(c) Consider use of a flow diagram	NA
Descriptive data	14*	(a) Give characteristics of study participants (eg demographic, clinical, social) and information on exposures and potential confounders	Table 1
		(b) Indicate number of participants with missing data for each variable of interest	NA
		(c) *Cohort study*—Summarise follow-up time (eg, average and total amount)	NA
Outcome data	15*	*Cohort study*—Report numbers of outcome events or summary measures over time	Table 1
		Case-control study—Report numbers in each exposure category, or summary measures of exposure	*NA*

		Cross-sectional study—Report numbers of outcome events or summary measures	*NA*
Main results	16	(*a*) Give unadjusted estimates and, if applicable, confounder-adjusted estimates and their precision (eg, 95% confidence interval). Make clear which confounders were adjusted for and why they were included	NA
		(*b*) Report category boundaries when continuous variables were categorized	Table 1
		(*c*) If relevant, consider translating estimates of relative risk into absolute risk for a meaningful time period	NA

Continued on next page

Other analyses	17	Report other analyses done—eg analyses of subgroups and interactions, and sensitivity analyses	NA
**Discussion**			
Key results	18	Summarise key results with reference to study objectives	Table 1
Limitations	19	Discuss limitations of the study, taking into account sources of potential bias or imprecision. Discuss both direction and magnitude of any potential bias	Table 1
Interpretation	20	Give a cautious overall interpretation of results considering objectives, limitations, multiplicity of analyses, results from similar studies, and other relevant evidence	Table 1
Generalisability	21	Discuss the generalisability (external validity) of the study results	Table 1
**Other information**			
Funding	22	Give the source of funding and the role of the funders for the present study and, if applicable, for the original study on which the present article is based	Table 1

*Give information separately for cases and controls in case-control studies and, if applicable, for exposed and unexposed groups in cohort and cross-sectional studies.

Note: An Explanation and Elaboration article discusses each checklist item and gives methodological background and published examples of transparent reporting. The STROBE checklist is best used in conjunction with this article (freely available on the Web sites of PLoS Medicine at http://www.plosmedicine.org/, Annals of Internal Medicine at http://www.annals.org/, and Epidemiology at http://www.epidem.com/). Information on the STROBE Initiative is available at www.strobe-statement.org.

Supplementary S2: Variable descriptions

Baseline characteristics included procedure type RLAR or non-restorative LAR (NRLAR)), nation (Netherlands, France, United Kingdom, Spain, Italy or Belgium), sex (male/female), age at time of surgery (years), ASA-classification (I, II, III, or IV), BMI (kg/m2), BMI categories (underweight (<18.5), normal weight (18.5-25.0), overweight (25.0-30.0), and obese (>30.0)), diabetic comorbidity (yes/no), cardiovascular comorbidity (yes/no), history of abdominal surgery (yes/no), preoperative T-staging (T1, T2, T3, or T4), preoperative N-staging (N0, N1, or N2), preoperative M-staging (M0 or M1), tumour height from the ARJ on MRI in cm, preoperative CRM-staging on MRI (negative or positive), preoperative extramural venous invasion (EMVI) staging on MRI (negative or positive), NAT (yes/no), type of NAT (none, only chemo, short-course radiotherapy, chemoradiation, chemoradiation + chemotherapy (total neoadjuvant therapy), or other), and year of surgery. Tumour location was determined using preoperative imaging (MRI pelvis or, if unavailable, CT-pelvis/endoscopy), using distance from the ARJ.

Intraoperative outcomes included conversion (yes/no, defined as any unplanned extension of the extraction site during surgery), conversion type (open, laparoscopic, strategic, or reactive), conversion reason (complications, exposure, narrow pelvis, extensiveness of tumour, access, or other), anastomosis performed (yes/no), stoma placed (yes/no), stoma type (ileostomy, diverting ileostomy, end ileostomy, colostomy, diverting colostomy, or end colostomy), intraoperative complications (yes/no), intraoperative bleeding (yes/no), intraoperative perforation (yes/no), other intraoperative complications (yes/no), operative time in minutes.

Pathological outcomes included pathological T-staging (pT0, pT1, pT2, pT3, or pT4), pathological N-staging (pN0, pN1, or pN2), pathological M-staging (pM0 or pM1), number of lymph nodes harvested, number of positive lymph nodes harvested, pathological CRM (negative (>1mm) or positive (≤1 mm)), completeness of resection (incomplete, nearly complete, or complete), and pathological resection quality (R0, R1, or R2). Pathological staging was modified according to the American Joint Committee on Cancer 8th edition staging system during data review.[38]

Postoperative outcomes included postoperative complications (yes/no), surgical postoperative complications (yes/no, defined as any surgery related complication), postoperative ileus (yes/no), postoperative wound infections (yes/no), postoperative bleeding (yes/no), type of postoperative complications (none, yes, abscess, fascia dehiscence, bowel perforation, leakage of bladder/ureter, other surgical complications, pulmonary, cardiac, thromboembolic, infectious, neurologic, urologic, other non-surgical complications), postoperative surgical complications with Clavien-Dindo classification[39] (none, grade I, grade

II, grade IIIa, grade IIIb, grade IV, grade IVa, grade IVb, grade V), anastomotic leakage (yes/no), anastomotic leakage time (none, early (<30 days), or late (>30 days)), anastomotic leakage grade (none, grade A, grade B, or grade C; according to ISREC (The International Study Group of Rectal Cancer) classification), anastomotic leakage treatment (none, medicine, radiological drainage of abscess, transanal drainage of abscess, endosponge, relaparotomy with deviating stoma, relaparotomy with reversal of anastomosis and end stomy, or other), reintervention within 31 days (yes/no), reintervention type (radiologic, laparoscopic, open surgery, transanal surgery, endoscopic, or other), readmission within 31 days (yes/no), LOS in days, adjuvant chemotherapy (yes/no), death (yes/no), cause of death (cancer related or not cancer related), lost to follow-up (yes/no), overall survival in months (OS), LR (yes/no), location of LR (anterior above, anterior below, inferior, central anastomotic, central non-anastomotic, posterior, lateral at right, lateral at left, or peritoneal reflection), time to LR in months, systemic recurrence (SR) (yes/no), location of SR (lung, liver, peritoneal, bone, ovaria, brain, or other), time to SR in months, disease-free survival (DFS) (yes/no), and DFS in months. SR was defined as any distant metastasis, either pathologically proven or suspect for recurrence on radiological imaging that showed growth on consecutive imaging. DFS was defined as the percentage of patients alive at follow-up without recurrent disease.
